# COVID-19 and Respiratory Failure: A Retrospective Observational Study From a Rural Midwest Hospital

**DOI:** 10.7759/cureus.47593

**Published:** 2023-10-24

**Authors:** Alex Kneller, Cyril Abadir, Osaheni Amadasu, Matias Matias, Robert D Arnce, Nova Beyersdorfer, Dennis W Wolff, Greg Stahl, Kerry Johnson, Scott Goade

**Affiliations:** 1 Medicine, Kansas City University, Joplin, USA; 2 Primary Care, Kansas City University, Joplin, USA; 3 Emergency Medicine, Freeman Health System, Joplin, USA; 4 Basic Sciences and Pharmacology, Kansas City University, Joplin, USA; 5 Statistics, Freeman Health System, Joplin, USA; 6 Mathematics, Missouri Southern State University, Joplin, USA; 7 Clinical Pharmacy, Freeman Health System, Joplin, USA

**Keywords:** midwest, mortality, respiratory failure, covid-19, hypercapnia, hypoxia, pulmonology

## Abstract

Background

Infection with severe acute respiratory syndrome coronavirus 2 (SARS-CoV-2) produces the coronavirus disease of 2019 (COVID-19), primarily presenting with respiratory symptoms, including cough, shortness of breath, etc. Respiratory failure can present similarly to a COVID-19 infection, and COVID-19 infection can cause respiratory failure. Thus, it is important to study respiratory failure, COVID-19, and the interaction between the two in hopes of improving patient outcomes. In this study, we compared mortality rates in patients admitted with COVID-19, respiratory failure, or both. Mortality rates in our study populations were further scrutinized based on patient age.

Materials and methods

Respiratory failure and COVID-19 data were collected via the electronic medical records system at Freeman Health System, a 410-bed, rural hospital, in Neosho and Joplin, Missouri, from April 2020 through December 2021. The patient population included all patients admitted to the hospital with a diagnosis of COVID-19 or respiratory failure, as defined by the International Classification of Disease, Tenth Revision (ICD-10). Patients with or without COVID-19, with or without respiratory failure, and patients with respiratory failure with COVID-19 were included.

Results

There was a significant increase in mortality (17.28%) in patients with COVID-19 and respiratory failure (P1) compared to patients with COVID-19 who did not have respiratory failure (P2). No significance was found when comparing patients with COVID-19 and respiratory failure (P1) and patients with respiratory failure without COVID-19 (P3) (p value=0.4921). In contrast, when divided based on age, we found a significant increase in mortality in patients 65 and older with COVID-19 and respiratory failure compared to patients 65 and older with respiratory failure who did not have COVID-19 (P5). There were no significant mortality increases in other comparisons.

Conclusion

When comparing patient populations within the Freeman Health System, patients with COVID-19 and respiratory failure had similar mortality rates as those with respiratory failure without COVID-19, while patients with only COVID-19 had a markedly reduced mortality rate, relatively. The higher mortality rates in patients with only respiratory failure when compared to patients with both respiratory failure and COVID-19 indicate that the presence of respiratory failure likely plays a bigger role in the inflammatory response that reduces one's chance of survival in this setting. Furthermore, age was shown to be a significant risk factor as patients aged 65 and older showed a greater mortality rate when patients had both COVID-19 and respiratory failure compared to patients with both conditions below the age of 65. The decrease in immune response that results in older patients is likely the largest contributing factor along with the increased likelihood of patients in this population also having more comorbidities, further decreasing the chance of survival. Future studies can investigate alternate treatment plans for patients aged 65 and older who are at higher risk of mortality with COVID-19 and respiratory failure.

## Introduction

The COVID-19 pandemic had a catastrophic impact on society globally, as one of the deadliest pandemics in modern history, being responsible for over six million deaths worldwide, and additionally causing widespread economic distress [1]. More research on the topic of COVID-19 is necessary for a better understanding of the management, treatment with or without RNA vaccines, and prevention of future pandemics [2,3]. The initial step in coronavirus infection is the binding of the coronavirus spike (S) protein to certain proteins on, most commonly, respiratory cell membranes [4]. The common route of transmission is via exposure to respiratory droplets from infected individuals, and infection can lead to SARS-CoV-2-induced pneumonia or some type of respiratory distress of varying severity [5,6]. The large inflammatory response in SARS-CoV-2-induced pneumonia is due to the overactivation of the immune system, resulting in a "cytokine storm" characterized by the excessive release of inflammatory cytokines, such as interleukin-6 (IL-6) and tumor necrotic factor-alpha (TNF-α) [7]. Further vascular damage results in numerous conditions, such as pulmonary edema, pulmonary fibrosis, and microthrombi formation, which can embolize and cause downstream effects, etc. The severe impact of COVID-19 on the respiratory system and associated mortality necessitates discussion and evaluation of respiratory failure, independently and in conjunction with COVID-19 infection [8].

Respiratory failure develops when there is inadequate oxygen delivery or removal of carbon dioxide from the body via the pulmonary system [9]. There are two types of respiratory failure that present similarly but have different mechanisms of action. Type I respiratory failure occurs when the respiratory system cannot provide enough oxygen, leading to hypoxemia [10]. Alveolar hypotension, low atmospheric pressure, gas exchange deficiency, and ventilation/perfusion mismatch can cause type I failure. Type II respiratory failure occurs when the respiratory system is unable to adequately remove carbon dioxide, leading to hypercapnia [11]. There are multiple etiologies of respiratory failure, including pathology of the upper or lower respiratory tracts, central and peripheral nervous systems, and the anatomical chest wall and muscles of respiration. Due to the numerous causes of respiratory failure, the etiology is difficult to accurately ascertain. Understanding the interaction between COVID-19 and respiratory failure as well as the differences in mortality is important to develop better treatment options in hopes of improving patient outcomes.

Multiple risk factors intensify the mortality rates of patients diagnosed with COVID-19 and/or respiratory failure, and one of the most prevalent factors is age [12]. Generally, as people grow older and older, the amount of health conditions rises along with the severity of said conditions, and the same applies to COVID-19 and respiratory failure [13]. It is well understood that our immune system declines in functionality the older we get, from the decline in immune mediator production to the declining ability to keep up with infection, and the rate of mortality significantly increases as we get older [14]. Therefore, applying this understanding to patients diagnosed with COVID-19 and respiratory failure is important to more accurately shift treatment methods in hopes of improving mortality rates. According to the Centers for Disease Control and Prevention (CDC), between July 2021 and June 2022, there was a marked increase in the mortality rate of patients diagnosed with COVID-19 who were above the age of 65, sometimes being twice as great dependent on the variant of the virus [15]. Respiratory failure only contributes to the increased mortality rate in COVID-19 patients aged 65 and older, and this significance is of particular interest due to the statistical significance of the data gathered, which will be further discussed in this study [16]. Furthermore, more health factors need to be considered as well from social determinants of health to other comorbidities as these are bound to impact the mortality rates seen. Ultimately, more research needs to be conducted on the correlation between the age of patients diagnosed with COVID-19 and/or respiratory failure and the mortality rates associated. However, this study is beneficial in adding to the current group of studies done on similar topics.

## Materials and methods

Study overview

This retrospective observational study was approved by the Institutional Review Board of Freeman Health System. The study was designed to investigate the relationship between respiratory failure and COVID-19 on mortality.

Study population

The data were collected from the electronic medical records system at Freeman Health System a rural hospital, in Neosho and Joplin, Missouri. The primary patient population included all patients above the age of 18 who were admitted to the hospital with a diagnosis of either COVID-19 or respiratory failure, as defined by the International Classification of Disease, Tenth Revision (ICD-10), diagnostic codes listed below (Table 1). As a retrospective observational study, there was no requirement to obtain informed consent from individual patients; therefore, all patients above the age of 18 who were discharged from Freeman Health System from April 2020 through December 2021 that met the inclusion criteria were included in the dataset.

**Table 1:** International Classification of Diseases, Tenth Revision (ICD-10), codes that apply to respiratory failure and COVID-19 COVID-19 = coronavirus disease of 2019.

ICD-10 COVID-19	
U071	COVID-19
ICD-10 RESPIRATORY FAILURE	
J9600	Acute respiratory failure, unspecified whether with hypoxia or hypercapnia
J9601	Acute respiratory failure with hypoxia
J9602	Acute respiratory failure with hypercapnia
J9610	Chronic respiratory failure, unspecified whether with hypoxia or hypercapnia
J9611	Chronic respiratory failure with hypoxia
J9612	Chronic respiratory failure with hypercapnia
J9620	Acute and chronic respiratory failure, unspecified whether with hypoxia or hypercapnia
J9621	Acute and chronic respiratory failure with hypoxia
J9622	Acute and chronic respiratory failure with hypercapnia
J9691	Respiratory failure, unspecified with hypoxia

**Figure 1:**
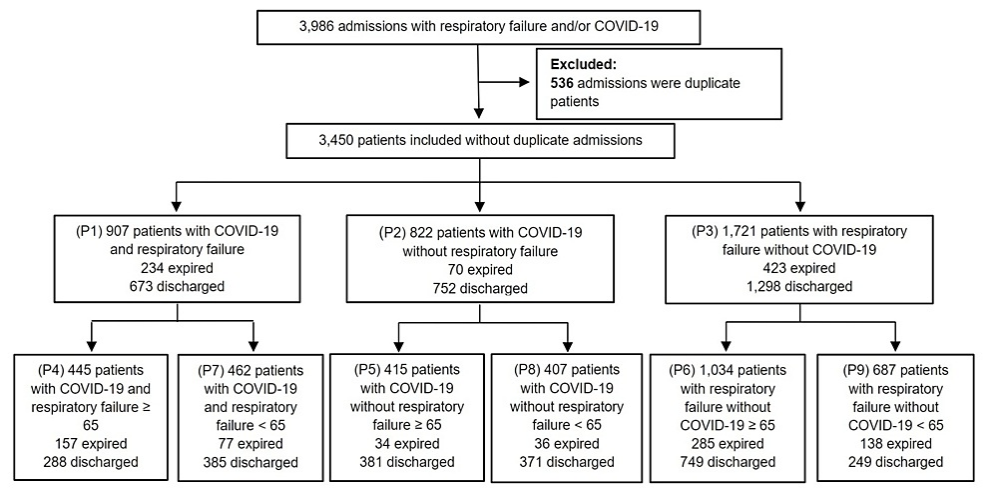
COVID-19 and respiratory failure patient population inclusion criteria P = Population

The primary patient population included 3,986 patients with either COVID-19, respiratory failure, or both conditions. Duplicate patient records were excluded from the data, leaving 3,450 patients. The primary patient population was divided into three subpopulations: patients with COVID-19 and respiratory failure, patients with COVID-19 without respiratory failure, and patients with respiratory failure who did not have COVID-19. Patients in each subgroup were then separated based on the outcome: discharge from the hospital or expiration (Figure 1). Each subgroup was also subdivided based on age for further comparison (Table 2).

**Table 2:** Defined populations comparing the association of COVID-19 and respiratory failure with respect to age COVID-19 = coronavirus disease of 2019.

Sample Populations	
P1	COVID-19 with Respiratory Failure
P2	COVID-19 without Respiratory Failure
P3	Respiratory Failure without COVID-19
P4	COVID-19 with Respiratory Failure ≥ 65
P5	COVID-19 without Respiratory Failure ≥ 65
P6	Respiratory Failure without COVID-19 ≥ 65
P7	COVID-19 with Respiratory Failure < 65
P8	COVID-19 without Respiratory Failure < 65
P9	Respiratory Failure without COVID-19 < 65

Statistical analysis

Two sample proportion tests were used to compare subgroups within the overall patient population. The first set of comparisons was focused on the initial three subgroups: patients with COVID-19 and respiratory failure (P1), patients with COVID-19 without respiratory failure (P2), and patients with respiratory failure who did not have COVID-19 (P3). Next, the three subgroups were compared using age as a variable. This study uses programmed formulas in Excel for all confidence intervals and p-values. The values reported are accurate to 15 decimals although results are rounded to four decimal places for readability. Finally, the programmed formulas were verified by the team's statistician.

## Results

The mortality of patients with COVID-19 and respiratory failure (P1) was 25.80%, while the mortality of patients with COVID-19 who did not have respiratory failure (P2) was 8.52%. This disparity in mortality percentages is relevant as the p-value is <0.0001, indicating statistical significance. This difference in mortality rates shows the effect that a coexisting diagnosis of respiratory failure has on patients who already have COVID-19. However, the mortality percentage of patients with respiratory failure who did not have COVID-19 (P3) was 24.58%. When comparing the mortality between P1 and P3, we see that there is not a significant difference as there is a p-value of 0.4921, showing that respiratory failure only has a significant impact on mortality when patients already have an existing diagnosis of COVID-19. Furthermore, there was a significant increase in mortality (17.28%) for patients with COVID-19 with a coexisting diagnosis of respiratory failure (P1) compared to patients with COVID-19 who did not meet the criteria for a diagnosis of respiratory failure (P2). When looking at age, there was no significant difference between mortality (P=0.1449) for patients with COVID-19 and a diagnosis of respiratory failure under the age of 65 (P7) and patients in the same age group with respiratory failure that did not have COVID-19 (P9) (Table 3).

**Table 3:** Mortality comparisons of patient populations with and without COVID-19 and respiratory failure P = population; CI = confidence interval P1 = COVID-19 with Respiratory Failure; P2 = COVID-19 without Respiratory Failure; P3 = Respiratory Failure without COVID-19; P4 = COVID-19 with Respiratory Failure ≥ 65; P5 = COVID-19 without Respiratory Failure ≥ 65; P6 = Respiratory Failure without COVID-19 ≥ 65; P7 = COVID-19 with Respiratory Failure < 65; P8 = COVID-19 without Respiratory Failure < 65; P9 = Respiratory Failure without COVID-19 < 65

Comparison	Population 1 Mortality	Population 2 Mortality	Population 1 vs Population 2	Lower 95% CI for P1-P2	Upper 95% CI for P1-P2	P-value
P1 vs P2	234 of 907	70 of 822	0.1728	0.1386	0.2071	<0.0001
	0.2580	0.0852				
P1 vs P3	234 of 907	423 of 1721	0.0122	-	-	0.4921
	0.2580	0.2458				
P2 vs P3	70 of 822	423 of 1721	0.1606	0.1327	0.1885	<0.0001
	0.0852	0.2458				
P2 vs P4	70 of 822	157 of 445	0.2677	0.2193	0.3160	<0.0001
	0.0852	0.3528				
P4 vs P5	157 of 445	34 of 415	0.2709	0.2192	0.3225	<0.0001
	0.3528	0.0819				
P4 vs P6	157 of 445	285 of 1034	0.0772	0.0251	0.1293	0.0029
	0.3528	0.2756				
P4 vs P7	157 of 445	77 of 462	0.1861	0.1302	0.2421	<0.0001
	0.3528	0.1667				
P4 vs P8	157 of 445	36 of 407	0.2644	0.2121	0.3166	<0.0001
	0.3528	0.0885				
P4 vs P9	157 of 445	138 of 687	0.1519	0.0984	0.2055	<0.0001
	0.3528	0.2009				
P5 vs P6	34 of 415	285 of 1034	0.1937	0.1558	0.2316	<0.0001
	0.0819	0.2756				
P5 vs P7	34 of 415	77 of 462	0.0847	0.0417	0.1278	0.0002
	0.0819	0.1667				
P5 vs P8	34 of 415	36 of 407	0.0065	-	-	0.7376
	0.0819	0.0885				
P5 vs P9	34 of 415	138 of 687	0.1189	0.0790	0.1589	<0.0001
	0.0819	0.2009				
P6 vs P7	285 of 1034	77 of 462	0.1090	0.0654	0.1525	<0.0001
	0.2756	0.1667				
P6 vs P8	285 of 1034	36 of 407	0.1872	0.1484	0.2259	<0.0001
	0.2756	0.0885				
P6 vs P9	285 of 1034	138 of 687	0.0748	0.0343	0.1152	0.0004
	0.2756	0.2009				
P7 vs P8	77 of 462	36 of 407	0.0782	0.0344	0.122	0.0006
	0.1667	0.0885				
P7 vs P9	77 of 462	138 of 687	0.0342	-	-	0.1449
	0.1667	0.2009				
P8 vs P9	36 of 407	138 of 687	0.1124	0.0717	0.1531	<0.0001
	0.0885	0.2009				

**Figure 2:**
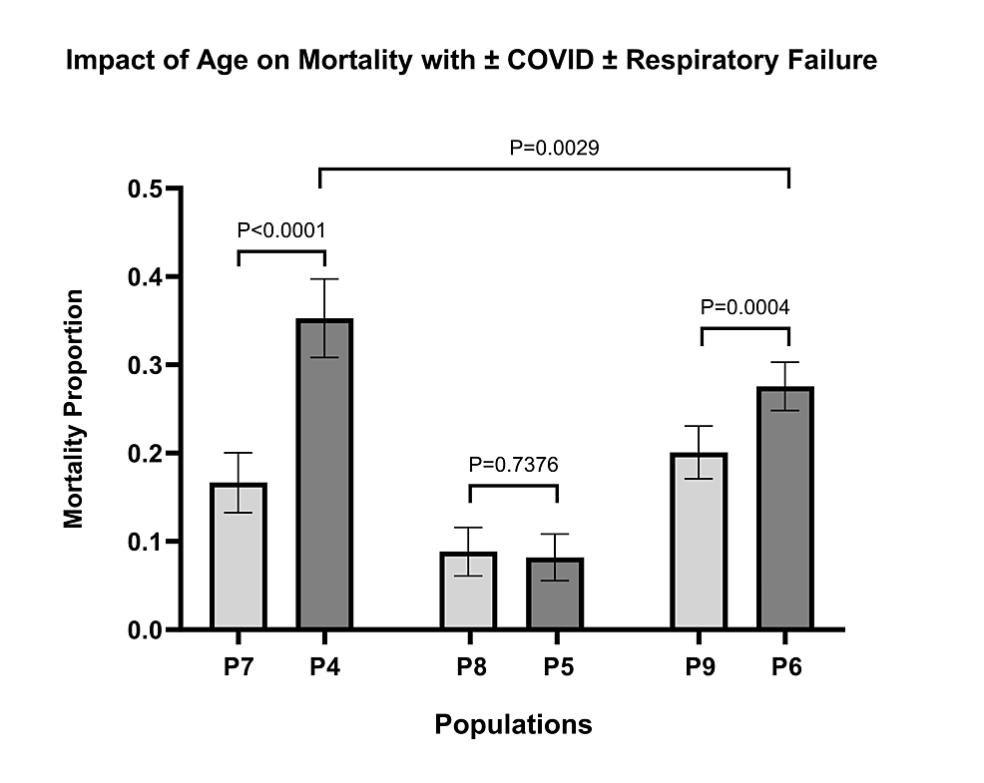
Impact of age on mortality ± COVID-19 ± respiratory failure COVID-19 = coronavirus disease of 2019 P4 = COVID-19 with Respiratory Failure ≥ 65; P5 = COVID-19 without Respiratory Failure ≥ 65; P6 = Respiratory Failure without COVID-19 ≥ 65; P7 = COVID-19 with Respiratory Failure < 65; P8 = COVID-19 without Respiratory Failure < 65; P9 = Respiratory Failure without COVID-19 < 65

The mortality due to age results showed a great significance when comparing COVID-19 with respiratory failure < 65 (P7) with COVID-19 with respiratory failure ≥ 65 (P4) (<0.000001). It was also noted that, when comparing P4 with P6, the statistical significance for mortality decreased (<0.0029). In addition, when contrasting COVID-19 without respiratory failure < 65 (P8) and COVID-19 without respiratory failure ≥ 65 (P5), there was no significant difference in mortality (<0.7376) (Figure 2).

A different study that occurred in Greece focused on the impact of age on mortality in patients with COVID-19 and found a significant increase in mortality based on age, with patients under the age of 65 having a 60-day survival rate of 89.3% and patients 65 and older having a 60-day survival rate of 58%. This study did not include the status of respiratory failure as a comorbidity but does indicate that our findings were not an isolated occurrence [17].

## Discussion

The initial purpose of the study was to determine the relationship between COVID-19 and respiratory failure. As expected, there was a significant increase in mortality (17.28%) for patients with COVID-19 with respiratory failure (P1) compared to patients with COVID-19 who did not meet the criteria for the diagnosis of respiratory failure (P2) (Table 3). However, there was no significant difference (P=0.4921) between patients with COVID-19 and respiratory failure (P1) and patients with respiratory failure without COVID-19 (P3). When looking at age, there was no significant difference between mortality (P=0.1449) for patients with COVID-19 and respiratory failure under the age of 65 (P7) and patients with respiratory failure without COVID-19 in the same age range (P9). For patients aged 65 and older, however, there was a greater incidence of mortality in patients with COVID-19 and respiratory failure compared to patients in the same age range with respiratory failure who did not have COVID-19 (Figure 2). Our results suggest that COVID-19 has a significant impact on patients aged 65 and older with respiratory failure but does not significantly impact patients younger than 65 with respiratory failure.

Although our data provided us with valuable conclusions, the study focuses more on the rural population for data analysis, instead of a more comprehensive population that contains suburban, city, and rural. Due to this, the results found in this study may not apply to populations outside the rural Midwest area. The population in the study was not randomly sampled, and it is unclear whether the samples were accurate representations of the respective populations. There were limitations on the control over socioeconomic variables, such as diversity, location, and access to healthcare that could have affected the overall analysis. There were insufficient sample sizes within the ICD-10 subclassifications of respiratory failure to make comparisons of the different forms of respiratory failure. The patient population from the study likely had additional underlying diseases, including various other comorbidities, such as hypoxia and hypercapnia. These comorbidities and diseases could have played a significant role in respiratory outcomes. Patients' past medical histories and medical profiles were not taken into account, which could hinder the understanding of how the conditions were managed.

## Conclusions

This observational study of patient populations of a rural Midwestern region revealed that patients with COVID-19 and respiratory failure did not have a significant difference compared to patients solely with respiratory failure. When adding another variable (age), the data suggest that patients aged 65 and older diagnosed with COVID-19 and respiratory failure had significantly higher mortality than patients under the age of 65 with the same diagnoses. COVID-19 alone did not increase the mortality of patient populations when comparing patients above and below the age of 65. The comorbidity of respiratory failure produces a significant difference between the age groups.

The data suggest that physicians need to critically integrate patient age into the decision-making process for the treatment and management of COVID-19 and respiratory failure. Future work on this topic may include alternate treatment plans for patients based on age or the differences in age-based outcomes for COVID-19 and respiratory failure in rural, suburban, and urban hospital settings. These further analyses, in conjunction with this work, will improve patient outcomes in rural settings and lend assistance to needed data to decrease the mortality of COVID-19 and pandemics in the future.
